# Nutritional status in young children prior to the malaria transmission season in Burkina Faso and Mali, and its impact on the incidence of clinical malaria

**DOI:** 10.1186/s12936-021-03802-2

**Published:** 2021-06-22

**Authors:** Mariken de Wit, Matthew Cairns, Yves Daniel Compaoré, Issaka Sagara, Irene Kuepfer, Issaka Zongo, Amadou Barry, Modibo Diarra, Amadou Tapily, Samba Coumare, Ismaila Thera, Frederic Nikiema, R. Serge Yerbanga, Rosemonde M. Guissou, Halidou Tinto, Alassane Dicko, Daniel Chandramohan, Brian Greenwood, Jean Bosco Ouedraogo

**Affiliations:** 1 London School of Hygiene and Tropical Medicine, London, UK; 2 Institut de Recherche en Sciences de La Santé, Bobo-Dioulasso, Burkina Faso; 3 Malaria Research and Training Centre, University of Science, Techniques, and Technologies of Bamako, Bamako, Mali

**Keywords:** Malaria, Chronic malnutrition, Acute malnutrition, Seasonal malaria chemoprevention, Burkina Faso, Mali

## Abstract

**Background:**

Malaria and malnutrition remain major problems in Sahel countries, especially in young children. The direct effect of malnutrition on malaria remains poorly understood, and may have important implications for malaria control. In this study, nutritional status and the association between malnutrition and subsequent incidence of symptomatic malaria were examined in children in Burkina Faso and Mali who received either azithromycin or placebo, alongside seasonal malaria chemoprevention.

**Methods:**

Mid-upper arm circumference (MUAC) was measured in all 20,185 children who attended a screening visit prior to the malaria transmission season in 2015. Prior to the 2016 malaria season, weight, height and MUAC were measured among 4149 randomly selected children. Height-for-age, weight-for-age, weight-for-height, and MUAC-for-age were calculated as indicators of nutritional status. Malaria incidence was measured during the following rainy seasons. Multivariable random effects Poisson models were created for each nutritional indicator to study the effect of malnutrition on clinical malaria incidence for each country.

**Results:**

In both 2015 and 2016, nutritional status prior to the malaria season was poor. The most prevalent form of malnutrition in Burkina Faso was being underweight (30.5%; 95% CI 28.6–32.6), whereas in Mali stunting was most prevalent (27.5%; 95% CI 25.6–29.5). In 2016, clinical malaria incidence was 675 per 1000 person-years (95% CI 613–744) in Burkina Faso, and 1245 per 1000 person-years (95% CI 1152–1347) in Mali. There was some evidence that severe stunting was associated with lower incidence of malaria in Mali (RR 0.81; 95% CI 0.64–1.02; p = 0.08), but this association was not seen in Burkina Faso. Being moderately underweight tended to be associated with higher incidence of clinical malaria in Burkina Faso (RR 1.27; 95% CI 0.98–1.64; p = 0.07), while this was the case in Mali for moderate wasting (RR 1.27; 95% CI 0.98–1.64; p = 0.07). However, these associations were not observed in severely affected children, nor consistent between countries. MUAC-for-age was not associated with malaria risk.

**Conclusions:**

Both malnutrition and malaria were common in the study areas, high despite high coverage of seasonal malaria chemoprevention and long-lasting insecticidal nets. However, no strong or consistent evidence was found for an association between any of the nutritional indicators and the subsequent incidence of clinical malaria.

**Supplementary Information:**

The online version contains supplementary material available at 10.1186/s12936-021-03802-2.

## Background

Malaria remains one of the most common infectious diseases worldwide, with an estimated 229 million cases in 2019 [1]. Improved use of existing control tools and new interventions are urgently needed in countries where progress in reducing the malaria burden has stalled [2]. Several of the countries that report an unchanged or increasing malaria burden lie within the Sahel region of West Africa [3], where malaria incidence peaks annually during the rainy season. These countries include Burkina Faso and Mali, where malaria is the leading cause of death in children under 5 years of age [4]. In Burkina Faso, malaria was responsible for 66% of deaths in children under 5 seen at a health facility in 2018 according to a survey from the Ministry of Health [5]. In such countries, seasonal malaria chemoprevention (SMC) is increasingly being used at scale to tackle the malaria burden. Another common health issue in young children living in the Sahel is undernutrition, which is responsible for the death of about 577,000 children in the Sahel region each year [6].

Malnutrition and malaria morbidity often co-occur and children severely affected by one are often affected by both. A pooled analysis from demographic and health survey (DHS) data in West Africa, which included some of the areas of highest malaria endemicity in all of Africa [7], showed that 10.0% of children under 5 years of age are wasted, 20.1% are underweight and 31.8% are stunted [8]. Malnutrition has long been known to be associated with infectious diseases [9], but its effect on malaria remains unclear. Interpreting associations is made challenging by the multifactorial causes of malnutrition, and the range of different metrics used to assess different aspects of nutritional status. A recent systematic literature review found no consistent association between malaria risk and malnutrition, although chronic malnutrition was associated with severity of malaria [10]. Another systematic review which reviewed nutritional status based on anthropometry and malaria infection, concluded that malnutrition does not have a large impact on malaria morbidity, but might negatively affect malaria mortality and severity [11]. Because of the possible bidirectional relationship between nutritional status and malaria, longitudinal cohort studies are the preferred design to disentangle these effects and reduce the impact of reverse causality. However, even among longitudinal studies, results are heterogeneous [12–15]. Only one study looked at the effect of a low mid-upper arm circumference (MUAC)-for-age, this showed an increased malaria risk in a sub-group of children who were under the age of 9 months [16]. Studies vary greatly in study population, location, sample size, presence of uncontrolled confounding and malaria epidemiology, making comparison across studies difficult.

Given current interest in incorporating nutritional screening or other nutritional interventions with SMC delivery [17], it is important to understand more about the nutritional status of children immediately prior to the rainy season, when SMC delivery begins. Clarifying the direct effect of malnutrition on malaria risk would help in understanding whether nutritional interventions might have the potential to assist with malaria control in the Sahel region. Finally, nutritional status may impact on the effectiveness of malaria control strategies, including SMC, affecting the ability of children to absorb medication, or the dose by weight that they receive, although this is poorly understood [10, 18].

Data from a clinical trial of SMC conducted between 2014 and 2016 in Burkina Faso and Mali was used to explore these issues. In addition to the more common nutritional indicators (stunting, wasting, underweight) this study also measured MUAC. MUAC was first thought to be relatively age and gender independent, but in 1993 a WHO Expert Committee recommended using MUAC-for-age because important age and gender-specific differences became apparent [19]. Little is known about the association between MUAC-for-age and malaria risk. As most similar studies to date have used measures of chronic malnutrition [11], this alternative measure of acute malnutrition is of specific interest. The association between nutritional status and malaria has never been studied in a longitudinal cohort of this size (n > 20,000). This study aims to establish the relationship between nutritional status shortly before the malaria transmission season and incidence of clinical malaria in young children aged 3–59 months during the malaria transmission season in Burkina Faso and Mali.

## Methods

### Study population

This study was conducted in Houndé district, Burkina Faso, and in Bougouni district, Mali as part of a large trial which studied the effect of adding azithromycin (AZ) to SMC (with sulfadoxine/pyrimethamine (SP) and amodiaquine (AQ)) on deaths and hospital admissions in young African children, as described in detail elsewhere [20]. A separate analysis indicated that the addition of AZ had no impact on nutritional indicators [21]. In brief, an initial household census was conducted prior to the rainy season in 2014, to recruit eligible children who would be aged between 3 and 59 months on 1 August, 2014. All children were given a long-lasting insecticide-treated bed net. Children were randomized by household to receive four 3-day courses of AZ or placebo alongside SMC with SP + AQ at monthly intervals during the peak malaria transmission season (August to November). All doses were based on age and administered by trial staff. Newly eligible children aged 3–59 months were recruited at an additional census conducted in May 2015 and May 2016. Children with a chronic disease, a known allergy to SP, AQ or AZ, or who were taking cotrimoxazole were excluded.

Nutritional status of the cohort was assessed in two ways: by assessment of MUAC in all study children in 2015 (Nutritional Cohort 1), intended to maximize the sample size available, and by a more detailed nutritional survey in 2016 (Nutritional Cohort 2), intended to explore nutritional status in more detail among a smaller number of children. No nutritional status measurements were taken prior to the 2014 rainy season. In 2015, MUAC was measured prior to the rainy season at the time of annual census in all study children within the age range 3–59 months, apart from 1253 children who were in follow-up, but who did not attend the screening visit. In 2016, a random sub-sample of approximately 2000 study children per country, stratified on randomization group and age, were selected for a more detailed anthropometric survey by an independent statistician. These children had MUAC, weight and height measured at the start and end of the 2016 transmission season.

### Intervention

Infants 3–11 months of age received a combined 250 mg of sulfadoxine and 12.5 mg of pyrimethamine plus 75 mg of AQ on day 1 and received 75 mg of AQ on days 2 and 3 (Guilin Pharmaceutical, Shanghai, China). In addition, they were randomly assigned to receive either 100 mg of AZ or matching placebo on days 1, 2, and 3 (Cipla, Mumbai, India). Children 1–4 years of age received double these doses. All doses were based on age and administration was directly observed by trial staff.

### Anthropometric measurements

Participants were weighed using a mechanical scale from SECA (Chino, CA, USA) with or without a tray, according to size, and weight was recorded to the nearest 0.1 kg. Recumbent length measurements were taken for children under 2 years of age and standing height measurements for children above 2 years using a stadiometer, recording the measurement to the nearest 0.1 cm. Length and height measurements were taken twice and the average was recorded. If the difference between the two measures was larger than 0.5 cm a third measure was taken and the two values with a difference smaller than 0.5 cm were used. MUAC was measured on the left arm to the nearest 0.1 cm with a MUAC tape.

### Morbidity surveillance

Morbidity episodes were recorded passively at study health centres and at the hospital in each district. In Mali, to avoid missing malaria episodes in villages located further from the health centres, malaria morbidity was also recorded by trained community health workers using the same procedures as described below. Children who presented to a community health worker or outpatient clinic at a health centre when they were feeling unwell, had a rapid diagnostic test (RDT) for malaria performed. Malaria diagnosis was confirmed when the child had a fever (temperature ≥ 37.5 °C, and/or a history of fever within the previous 24 h) and a positive RDT for malaria or a positive blood smear (as described below). Free first-line treatment (artemether-lumefantrine) was offered on the basis of the RDT result. Blood smears were also taken from a systematic sample of study children (1 day per week in Burkina Faso and 1 week per month in Mali) for quality control of the RDT, and read by two readers. Slides which were discordant for either positivity or parasite density were read by a third reader, and the discrepancy resolved following the algorithm developed by Swysen et al. [22].

### Data collection and management

Data on morbidity episodes were recorded on case report forms and entered into an electronic database by two separate data entry officers using the DataFax system. To avoid double counting of multiple attendances for the same clinical episode, malaria episodes within 7 days of a previous episode were discounted. Person-time at risk and malaria episodes were included up to the date of censoring for children who exited the study or who died during the study period.

### Statistical analysis

The number of children available for the 2015 MUAC survey was determined by the number recruited to the main trial, which was powered to detect a 25% difference with 90% power between the two study groups in the incidence of the primary trial end-point, death or hospital admission [20]. The required sample size for cross-sectional surveys (pre-season and post-season) in 2016 was driven by an outcome not analysed in the present study: the prevalence of SP and AQ resistance genotypes among study children. However, given the high incidence rate of malaria in the study area, a sample size of approximately 2000 children in each country from whom anthropometric measurements were taken in 2016 provided a high level of statistical power to detect even small differences in incidence rates according to pre-season nutritional status.

Different forms of malnutrition were evaluated in this study. Acute malnutrition was measured using weight-for-height (wasting), MUAC, and MUAC-for-age. Chronic malnutrition was measured using height-for-age (stunting). Being underweight (low weight-for-age) is a mixture of both chronic and acute malnutrition [23]. Nutritional indicators were calculated using standardized reference charts according to WHO guidelines [24, 25]. Weight-for-height was calculated for children above 65 cm, weight-for-length was calculated for children under 65 cm and this was combined into one weight-for-height/length variable.

A height-for-age Z-score below − 2 indicates stunting, a weight-for-age score below − 2 indicates underweight and a weight-for-height/length score below − 2 indicates wasting. Severity of nutritional indicators (stunting, wasting, underweight, low MUAC-for-age) was categorized into three groups: normal (z > − 2), moderately affected (− 2 < z < − 3), and severely affected (z < − 3).

Children with incomplete anthropometric data were included for the indicators that could be calculated, e.g., if weight, MUAC and age were collected, but height was missing, then weight-for-age and MUAC-for-age were calculated, but height-for-age and weight-for-height/length were not. Distributions of demographic and nutritional status variables were described as present prior to the transmission season. Distributions of z-scores for nutritional measures were compared between countries using the Wilcoxon rank-sum test. Prevalence of malnutrition according to each nutritional indicator was calculated by age and country. Associations between nutritional indicators and variables of interest were studied by creating logistic regression models with normally distributed random effects to adjust for clustering at the household level. Reliability of the estimates was checked by performing the quadrature check. Associations between nutritional status and malaria incidence were estimated using separate random effects Poisson models for each nutritional indicator adjusting for clustering in households and potential confounders. Variables which showed an association with both nutritional status and malaria incidence were considered to be potential confounders and were therefore included in the models. To be able to adjust for dosage as a covariate, the received dose was calculated by dividing the amount of drug administered by the child’s weight. For SP, less than 25 mg/kg was defined as below target and more than 70 mg/kg was defined as above target [26]. For AQ, less than 10 mg/kg was defined as below target and more than 15 mg/kg was defined as above target [27]. The dose of AZ was not investigated as a confounder because this was found to have no effect on malaria incidence in the main trial [20]. Intervention arm and SMC dose were included a priori because of their hypothesized effect on malaria risk. ‘Adjusted’ models included adjustment for age, gender, calendar month, intervention arm, and distance to health facility. ‘Fully adjusted’ models also included adjustment for SMC dose to be able to separately study its effect. Gender was a priori considered as potential effect modifier. Therefore, interaction between gender and nutritional status was examined by adding interaction terms to the multivariable model. All analyses were done in Stata/IC 16.

## Results

### Study population

A total of 20,185 children were included in the analysis in 2015 (9,889 in Burkina Faso and 10,296 in Mali). The sub-sample for which anthropometric measurements were obtained in 2016 comprised 4,149 children (2098 in Burkina Faso and 2051 in Mali). The distribution of baseline and nutritional variables is presented in Table 1. Participants were equally distributed by gender and intervention arm. In Mali, 45.7% of children were aged between 13 and 36 months in both years. This percentage was similar in Burkina Faso: 44.3% in 2015 and 44.7% in 2016. Distance to nearest health facility was smaller in Mali with 43.9–44.6% living within 1 km of the nearest health facility compared to 17.8–20.6% in Burkina Faso.

**Table 1:** Distribution of baseline variables

	Aug-15		Aug-16	
	N = 20,185		N = 4149	
	Burkina Faso	Mali	Burkina Faso	Mali
	N = 9889	N = 10,296	N = 2098	N = 2051
	Number (%)		Number (%)	
General characteristics				
Gender				
Boy	5087 (51.4)	5290 (51.4)	1095 (52.2)	1126 (54.9)
Girl	4802 (48.6)	5006 (48.6)	1003 (47.8)	925 (45.1)
Age in months				
12-Mar	1540 (16.0)	1387 (14.6)	283 (13.7)	304 (15.4)
24-Dec	2151 (22.3)	2242 (23.6)	448 (21.7)	415 (21.0)
24–36	2124 (22.0)	2095 (22.1)	475 (23.0)	487 (24.7)
36–48	1956 (20.2)	2044 (21.5)	449 (21.7)	395 (20.0)
48 +	1886 (19.5)	1733 (18.2)	411 (19.9)	371 (18.8)
Number missing	232	795	32	79
Intervention arm				
Placebo	4907 (49.6)	5192 (50.4)	1036 (49.4)	1037 (50.6)
AZ	4982 (50.4)	5104 (49.6)	1062 (50.6)	1014 (49.4)
Distance to health facility				
< 1 km	1759 (17.8)	4524 (43.9)	433 (20.6)	915 (44.6)
1–4 km	6,044 (61.1)	1608 (15.6)	1212 (57.8)	307 (15.0)
5–9 km	1908 (19.3)	1737 (16.9)	426 (20.3)	345 (16.8)
10 + km	178 (1.8)	2427 (23.6)	27 (1.3)	484 (23.6)
Nutritional status measures				
Height-for-age				
Median z-score [IQR]	–	–	− 1.2 [− 2.0- − 0.4]	− 1.2 [− 2.1- − 0.1]
Mildly stunted (− 2 < z < − 3)			356 (17.2)	335 (17.1)
Severely stunted (z < − 3)			164 (7.9)	204 (10.4)
Number missing			32	91
Weight-for-age				
Median z-score [IQR]	–	–	− 1.2 [− 2.2- − 0.4]	− 1.0 [− 1.9- − 0.3]
Mildly underweight (− 2 < z < − 3)			366 (17.7)	302 (15.4)
Severely underweight (z < − 3)			265 (12.8)	128 (6.5)
Number missing			32	91
Weight-for-height/length				
Median z-score [IQR]	–	–	− 0.9 [− 2.0- 0.0]	− 0.6 [− 1.5- 0.1]
Mildly wasted (− 2 < z < − 3)			260 (12.4)	186 (9.1)
Severely wasted (z < − 3)			292 (13.9)	131 (6.4)
Number missing			0	12
MUAC				
Median MUAC in mm [IQR]	145 [136–152]	145 [135–155]	145 [136–152]	145 [139–155]
Moderately low MUAC (125-135 mm)	1762 (17.8)	1781 (17.6)	395 (18.8)	310 (15.2)
Severely low MUAC (< 125 mm)	578 (5.8)	768 (7.6)	112 (5.3)	105 (5.2)
Number missing	0	147	0	14
MUAC-for-age				
Median z-score [IQR]	− 0.8 [− 1.4 to − 0.1]	− 0.7 [− 1.4 to 0.0]	− 0.8 [− 1.4 to − 0.2]	− 0.6 [− 1.2 to 0.0]
Low MUAC-for-age (− 2 < z < − 3)	807 (8.4)	745 (8.0)	145 (7.0)	112 (5.7)
Very low MUAC-for-age (z < − 3)	126 (1.3)	184 (2.0)	28 (1.4)	21 (1.1)
Number missing	334	924	42	93

Both cohorts (2015 and 2016) are presented with their nutritional measurements

### Prevalence of malnutrition

**Fig. 1:**
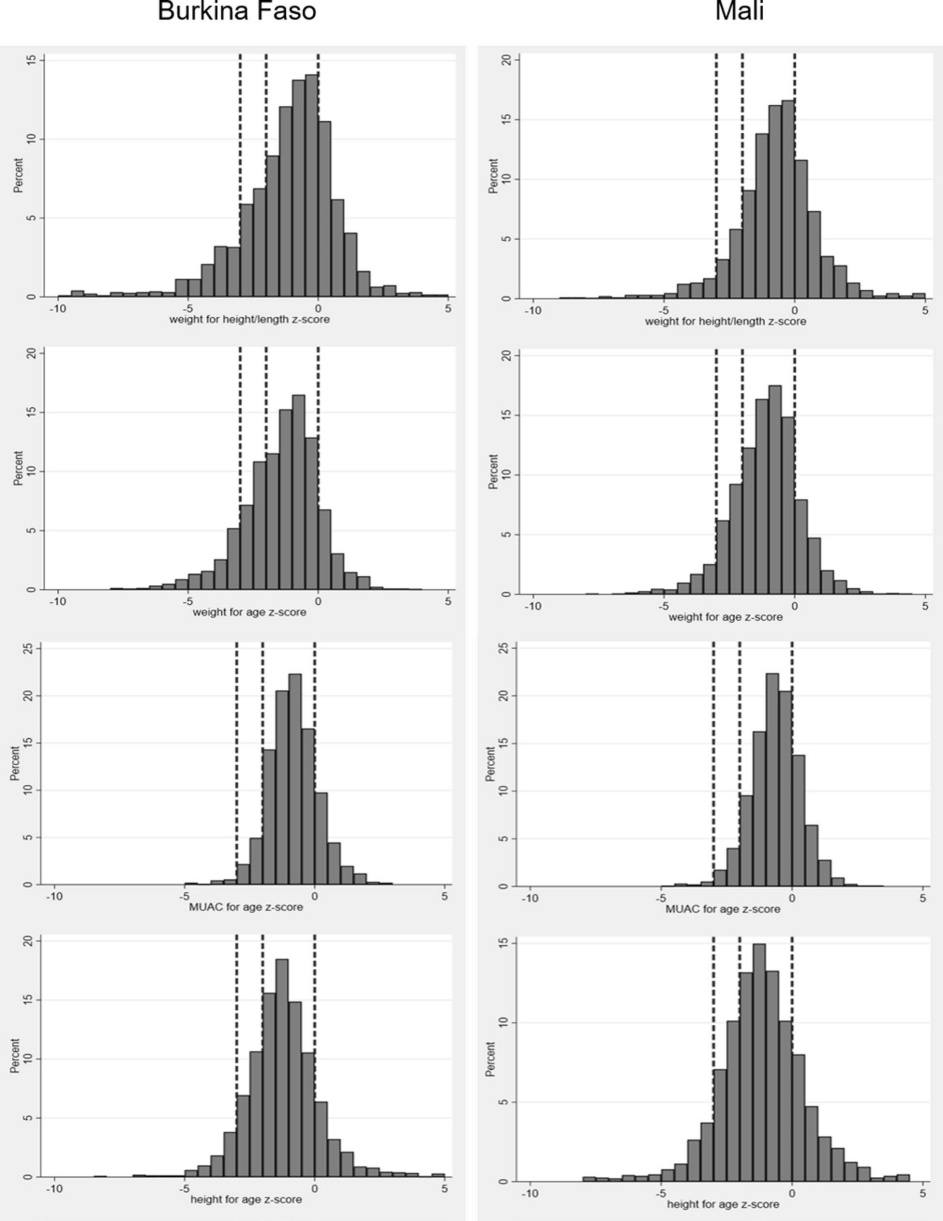
Histograms of the z-score distributions for weight-for-height/length (wasting), weight-for-age (underweight), MUAC-for-age and height-for-age (stunting) in 2016. Vertical lines show z-scores of 0, − 2 and − 3 as these indicate the severity categorization and null value. The left panel represents data from Burkina Faso, the right panel for Mali. Only values within a plausible range (− 10 to 5) are included

Poor nutritional status was common prior to the rainy season in 2016. Figure 1 shows histograms of z-scores for stunting, wasting, underweight and low MUAC-for-age for both countries in 2016. The dashed lines shows the cut-off values for the severity categories used for malnutrition (normal is z-score above − 2; mild malnutrition is a z-score − 2 to − 3; severe malnutrition is a z-score > − 3). This shows that nutritional z-scores were approximately normally distributed around a negative z-score. The median z-scores were below 0 for all nutritional measures in both countries and lowest for height-for-age (median z-score − 1.2 in both countries). Apart from for height-for-age (p = 0.2), there was very strong evidence that nutritional status z-scores were lower in Burkina Faso than in Mali in 2016 for all other measures (p < 0.001).

**Fig. 2:**
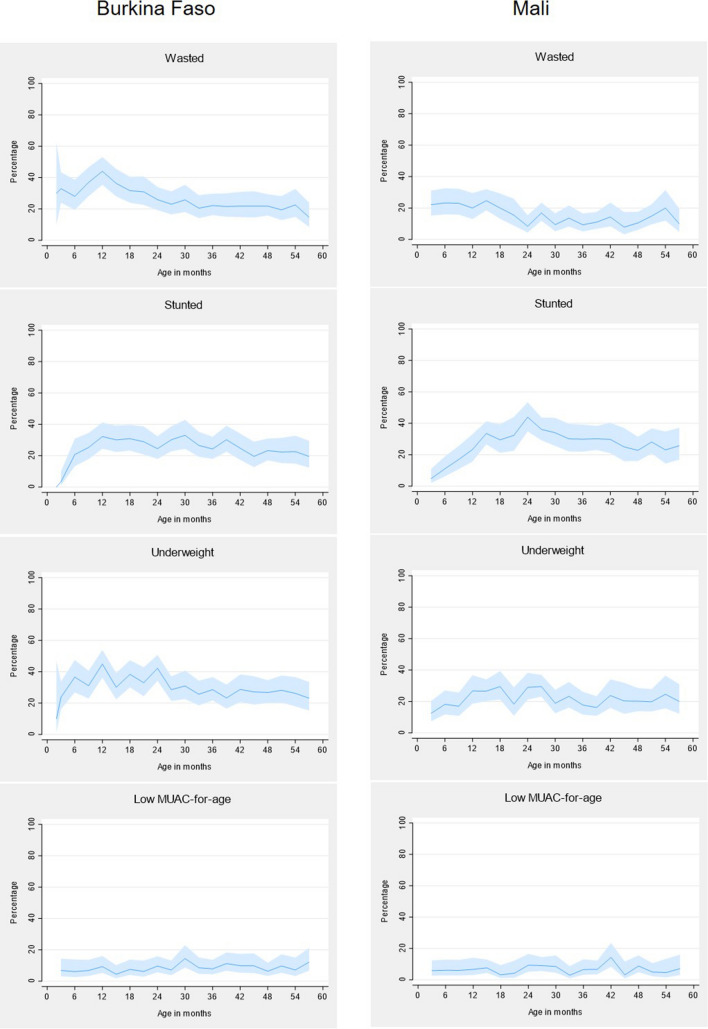
Variation in prevalence of malnutrition (z-score below − 2) in 2016 for different measures of nutritional status by age

The most prevalent form of malnutrition in Burkina Faso was being underweight, which affected 30.5% of children (95% CI 28.6–32.6). However, stunting was the most prevalent form of malnutrition in Mali affecting 27.5% of children (95%CI 25.6–29.5). Prevalence of malnutrition by age is shown in Fig. 2. The prevalence of low MUAC-for-age is lower than the other three nutritional measures in most age groups.

Using MUAC without adjusting for age and gender greatly overestimated the prevalence of malnutrition in younger age groups compared to the adjusted MUAC measure (see Additional file 1). MUAC-for-age was therefore used for all subsequent analyses. The correlation between the two main measure of acute malnutrition, MUAC-for-age and weight-for-height/length showed an r-squared of 0.10. When calculated for each year of age, results varied between 0.10 and 0.14.

### Factors associated with poor nutritional status

Univariate analyses (comparing children with poor nutritional status, z-score < − 2, to well-nourished children, z-score > − 2)) showed that drug doses were consistently associated with all nutritional indicators, see Table 2 and Additional file 2. Country-specific results can be found in Additional files 3 and 4. Information on dose of anti-malarials given was not available in 2015 because the weight of children was not measured. Children with a low weight-for-age prior to the transmission season were more likely to subsequently receive a dose of SMC drugs above the target of 70 mg/kg for SP and 15 mg/kg for AQ, a consequence of the fact that SP + AQ was dosed by age and not by body weight in this study. This was also observed for other measures of nutritional status. The only variable without evidence for an association with any nutritional indicator was intervention arm (i.e., receipt of AZ compared to placebo alongside the SMC regimen) (0.25 < p < 0.84). Age was associated with all nutritional indicators, apart from low MUAC-for-age in 2016. There is strong evidence that children in Mali have lower odds of wasting (OR = 0.43; 95% CI 0.35–0.54; p < 0.0001) and underweight (OR = 0.58; 95% CI 0.49–0.70; p < 0.0001) compared to children in Burkina Faso. The odds for low MUAC-for-age were similar between the two countries in 2015, but in 2016 odds were lower for children in Mali compared to Burkina Faso (OR = 0.77; 95% CI 0.59–0.99; p = 0.05). Children living further away from the health facility tended to have worse nutritional status compared to children living within 1 km of the nearest health facility.

**Table 2:** Association of baseline variables with stunting, wasting and underweight in 2016

	Stunted			Wasted			Underweight		
	Number (%)	Odds ratio	P-value	Number (%)	Odds ratio	P-value	Number (%)	Odds ratio	P-value
Gender			< 0.0001			0.11			0.01
Boy	629 (29.2)	1		488 (22.0)	1		606 (28.1)	1	
Girl	430 (23.0)	0.71 (0.61–0.83)		381 (19.8)	0.86 (0.70–1.04)		455 (24.3)	0.89 (0.68–0.94)	
Age in months			< 0.0001			< 0.0001			< 0.0001
3–12	79 (13.5)	1		162 (27.7)	1		132 (22.5)	1	
13–24	262 (30.4)	3.07 (2.26–4.18)		248 (28.8)	1.04 (0.76–1.42)		272 (31.6)	1.72 (1.29–2.28)	
25–36	308 (32.2)	3.38 (2.48–4.54)		174 (18.2)	0.48 (0.35–0.67)		277 (28.9)	1.50 (1.13–1.98)	
37–48	226 (26.9)	2.58 (1.88–3.48)		140 (16.7)	0.41 (0.29–0.58)		195 (23.2)	1.05 (0.79–1.40)	
48 +	184 (23.6)	2.09 (1.53–2.85)		132 (16.9)	0.44 (0.31–0.63)		185 (23.7)	1.10 (0.82–1.47)	
Country			0.11			< 0.0001			< 0.0001
Burkina Faso	520 (25.2)	1		552 (26.3)	1		631 (30.5)	1	
Mali	539 (27.5)	1.13 (0.97–1.32)		317 (15.6)	0.43 (0.35–0.54)		430 (21.9)	0.58 (0.49–0.70)	
Intervention arm			0.25	447 (21.6)		0.28			0.34
Placebo	547 (27.1)	1		422 (20.4)	1		545 (27.0)	1	
AZ	512 (25.5)	0.91 (0.79–1.07)			0.89 (0.73–1.10)		516 (25.7)	0.92 (0.78–1.09)	
Distance to health facility			0.004			< 0.0001			< 0.0001
< 1 km	341 (26.2)	1		194 (14.4)	1		253 (19.5)	1	
1–4 km	348 (23.5)	0.86 (0.71–1.04)		371 (24.5)	2.24 (1.73–2.90)		426 (28.7)	1.78 (1.44–2.20)	
5–9 km	220 (29.4)	1.18 (0.95–1.47)		191 (24.8)	2.35 (1.72–3.20)		244 (32.6)	2.22 (1.72–2.86)	
10 + km	150 (30.4)	1.25 (0.97–1.61)		113 (22.3)	1.86 (1.31–2.64)		138 (27.9)	1.66 (1.25–2.21)	
SP dose (mg/kg)			< 0.0001			< 0.0001			
< 25	2 (6.9)	0.20 (0.04–0.87)		1 (3.2)753 (18.9)	0.12 (0.01–1.01)		0 (0.0)	*Not relevant*^*a*^	
25–70	986 (25.5)	1		115 (89.8)	1		934 (24.1)		
> 70	71 (55.9)	4.26 (2.82–6.43)			89.5 (39.7–202)		127 (100.0)		
AQ dose (mg/kg)			< 0.0001			< 0.0001			< 0.0001
< 10	42 (5.5)	0.15 (0.10–0.21)		33 (4.2)	0.18 (0.12–0.26)		1 (0.1)	0.004 (0.001–003)	
10–15	605 (25.7)	1		423 (17.4)	1		506 (21.5)	1	
> 15	412 (45.4)	2.68 (2.20–3.37)		413 (45.3)	5.16 (4.00–6.67)		554 (61.1)	7.05 (5.38–9.25)	

Number and proportion with z < − 2 are shown together with odds ratios and p-values. Likelihood ratio test p-values are presented to indicate a global measure of association

^a^OR cannot be calculated as prevalence is 0% and 100% in the non-reference groups

### Relationship between malnutrition and malaria incidence

In Burkina Faso, the rate of malaria episodes in 2015 (1454 per 1000 person-years; 95% CI 1407–1501) was higher than in 2016 (675 per 1000 person-years; 95% CI 613–744). In Mali, the rate was lower in 2015 (1042 per 1000 person-years; 95% CI 1002–1083) compared to 2016 (1245 per 1000 person-years; 95% CI 1152–1347).

After full adjustment, there is little evidence of an association with any of the nutritional indicators (Table 3). Moderately underweight children in Burkina Faso seemed to be at a 1.3 times increased odds of clinic malaria (OR 1.27; 95% CI 0.98–1.64; p = 0.07). In Mali, moderately wasted children seemed to be at a 1.3 times increased odds of clinic malaria (OR 1.27; 95% CI 0.98–1.64; p = 0.07). Both of these effects were however not observed in severely affected children nor in the other country. The strongest protective association found was for severe stunting in Mali (RR = 0.81; 95% CI 0.64–1.01; p = 0.08).

**Table 3:** Association between nutritional status and malaria incidence

	No of episodes	Rate per 1000 person-years	Unadjusted	Adjusted^a^	Fully adjusted^b^	Adjusted P-value
			Rate ratio (95% CI)	Rate ratio (95% CI)	Rate ratio (95% CI)	
Burkina Faso						
Height-for-age						
Normal	274	685 (608–771)	1	1	1	
Moderately stunted	126	729 (612–868)	1.05 (0.84–1.31)	1.11 (0.89–1.39)	1.08 (0.86–1.36)	0.49
Severely stunted	62	550 (429–706)	0.78 (0.58–1.04)	0.88 (0.65–1.18)	0.86 (0.63–1.17)	0.34
Mali						
Height-for-age						
Normal	491	1318 (1207–1440)	1	1	1	
Moderately stunted	196	1252 (1089–1440)	0.95 (0.80–1.13)	0.94 (0.79–1.12)	0.94 (0.78–1.13)	0.53
Severely stunted	119	1033 (863–1236)	0.78 (0.63–0.96)	0.80 (0.64–0.99)	0.81 (0.64–1.02)	0.08
Burkina Faso						
Weight-for-age						
Normal	281	654 (581–735)	1	1	1	
Moderately underweight	114	859 (715–1032)	1.29 (1.02–1.62)	1.29 (1.02–1.63)	1.27 (0.98–1.64)	0.07
Severely underweight	67	545 (429–692)	0.83 (0.63–1.10)	0.94 (0.71–1.25)	0.94 (0.66–1.34)	0.75
Mali						
Weight-for-age						
Normal	578	1277 (1177–1286)	1	1	1	
Moderately underweight	165	1314 (1128–1531)	1.02 (0.85–1.23)	1.05 (0.87–1.26)	1.07 (0.88–1.32)	0.48
Severely underweight	63	951 (743–1218)	0.74 (0.56–0.97)	0.77 (0.58–1.01)	0.82 (0.60–1.13)	0.23
Burkina Faso						
Weight-for-height/length						
Normal	353	688 (619–763)	1	1	1	
Moderately wasted	58	674 (521–872)	0.98 (0.72–1.32)	1.04 (0.77–1.40)	1.00 (0.73–1.36)	1
Severely wasted	58	599 (463–774)	0.88 (0.65–1.18)	0.94 (0.69–1.26)	0.91 (0.66–1.27)	0.59
Mali						
Weight-for-height/length						
Normal	714	1260 (1171–1356)	1	1	1	
Moderately wasted	84	1376 (1111–1704)	1.02 (0.85–1.23)	1.21 (0.94–1.56)	1.27 (0.98–1.64)	0.07
Severely wasted	49	1142 (864–1512)	0.74 (0.56–0.97)	0.96 (0.70–1.31)	1.03 (0.74–1.43)	0.85
Burkina Faso						
MUAC-for-age (2016)						
Normal	417	690 (627–760)	1	1	1	
Low MUAC-for-age	34	643 (459–899)	0.95 (0.65–1.38)	0.96 (0.66–1.40)	0.95 (0.65–1.38)	0.78
Very low MUAC-for-age	7	636 (303–1333)	0.95 (0.43–2.11)	0.92 (0.42–2.05)	0.92 (0.41–2.04)	0.84
Mali						
MUAC-for-age (2016)						
Normal	726	1253 (1165–1348)	1	1	1	
Low MUAC-for-age	42	1070 (791–1448)	0.86 (0.62–1.20)	0.85 (0.61–1.18)	0.88 (0.63–1.23)	0.47
Very low MUAC-for-age	18	1929 (1216–3,062)	1.37 (0.82–2.31)	1.36 (0.81–2.29)	1.46 (0.86–2.47)	0.16
Burkina Faso						
MUAC-for-age (2015)						
Normal	4042	1490 (1445–1537)	1	1		
Low MUAC-for-age	421	1424 (1294–1567)	0.96 (0.86–1.07)	0.95 (0.86–1.06)		0.39
Very low MUAC-for-age	68	1437 (1133–1823)	0.97 (0.75–1.25)	1.02 (0.79–1.31)		0.88
Mali						
MUAC-for-age (2015)						
Normal	2791	1029 (992–1068)	1	1		
Low MUAC-for-age	297	1065 (950–1193)	1.03 (0.91–1.17)	1.02 (0.90–1.16)		0.77
Very low MUAC-for-age	91	1306 (1063–1604)	1.24 (0.99–1.55)	1.20 (0.96–1.51)		0.12

^a^Adjustment was made for age, gender, intervention arm, calendar month, and distance to health facility

^b^Additional adjustment was made for AQ dose for the 2016 variables (in addition to the variables listed above). No adjustment was made for SP dose to avoid collinearity

The association between MUAC-for-age and malaria was similar in 2015 and in 2016 in Mali, suggestive of an increased risk in those with very low MUAC-for-age (fully adjusted RR = 1.46 (95% CI 0.86–2.47) in 2016 and adjusted RR = 1.20 (95% CI 0.96–1.51) in 2015, although the confidence intervals overlapped unity in both years. No interaction between gender and nutritional status was found.

## Discussion

In this study, the nutritional status of young children was examined prior to the rainy season when SMC is administered. Nutritional status was generally poor in both countries in both years of the study. Prevalence varied for different forms of malnutrition, with underweight being the most prevalent form in Burkina Faso and stunting being the most common in Mali. Median z-scores ranged from − 0.6 to − 1.2, indicating that malnutrition is common among the whole study population instead of there being a small group of severely affected children. This can also be concluded from the approximate normal distribution of z-scores for the nutritional indicators. Although less frequent, high z-scores (above + 2) also occurred, suggesting a potential dual burden of over- and underweight in these regions. As it has been reported elsewhere [20], malaria incidence among the study children remained very high despite high coverage with SMC and provision of insecticidal nets.

Malaria incidence differed between the two countries in the different years of the study, reflecting seasonal variation in malaria transmission, and some differences in the timing of SMC administration with respect to the seasonal peak in transmission [28]. However, malaria incidence remained high in both study areas throughout the study period. Prevalence of malaria among school-age children not included in these analyses was above 50% at the end of the 2015 and 2016 rainy season in both countries [20]. No strong evidence was found for an association between any of the nutritional indicators and malaria. Severe chronic malnutrition tended to decrease the risk of a symptomatic malaria episode in Mali but not in Burkina Faso. Moderately underweight children in Burkina Faso and moderately wasted children in Mali seemed to be at an increased risk for clinical malaria, but these associations were not observed in severely affected children nor in the other country and may have been due to chance. Acute malnutrition did not affect malaria risk when measured as MUAC-for-age. A priori, it was expected that part of any association between nutritional status and malaria incidence might be explained by its effect on SMC dose (i.e., adjustment for dose might reduce the apparent effects of nutrition). A large number of children received a dose of AQ above the recommended threshold of 15 mg/kg/day. However, adjustment for AQ dose did not alter the associations between malnutrition and malaria risk, suggesting that receipt of a dose of AQ above this threshold was not associated with increased protection from malaria.

Prevalence estimates of malnutrition found in the current study were different to DHS estimates in the same regions [29, 30]. The prevalence of stunting was 5–12% lower in this study compared to DHS in both countries. Estimates of underweight and wasting in Mali differed less than 5% from the DHS estimates. However, in Burkina Faso, DHS estimates of underweight and wasting were 10–15% lower than in this study. DHS data were collected 3–6 years before this study and at a different time of year, potentially explaining some of the observed differences.

Nutritional status does not appear to have a consistent impact on malaria risk when SMC is provided. This study provides some evidence of a protective effect of severe stunting on malaria risk in Mali. One other study also found a protective effect of stunting [31], some other studies showed an increased risk in stunted children [13, 14] or no association [12, 15, 32]. A systematic review of observational studies shows that most studies suggested no association with malaria incidence [11]. Results presented in this study indicated a 27% increase in malaria risk for those moderately underweight in Burkina Faso, but no effect in severely underweight children and not in Mali. The absence of a clear association is supported by several other studies finding no consistent association [12–14, 32]. This study also showed a 27% increase in malaria risk for moderately wasted children in Mali. Also here, this effect was not seen in severely affected children and not in Burkina Faso. Most other studies showed no association between wasting and malaria [14, 15, 32], although one reported a protective effect [12].

Results from this study showed no effect of low MUAC-for-age on malaria risk, despite measurement of MUAC in a large number of children in 2015. However, another study looking at MUAC-for-age showed a 2.3-fold increase in odds of malaria for malnourished children under 9 months old [16]. MUAC-for-age was originally created as a more practical alternative for weight-for-height/length and is less prone to measurement error. It has been shown before that both measures partly identify different children as being malnourished (i.e., have different sensitivity and specificity) [33]. Studies indicate that MUAC has a better predictive ability for increased mortality risk than weight-for-height [34]. It is important to explore this relationship further and confirm these findings in other studies, since this is one of the very first studies to look at MUAC-for-age.

For none of the nutritional measures has any consistent association been found in the literature between malaria incidence and malnutrition, as in this study. This likely reflects the complexity of causes of malnutrition and the fact that it can be defined and measured in several ways [11]. Moreover, prevalence of malnutrition and incidence of malaria vary greatly by region possibly contributing to the contradictory results individual studies have found. People in different regions have different diets that especially affects micronutrient intake. The role of micronutrients in malaria is still controversial [35]. Additionally, the timing of the peak malaria season and of food shortage before harvest varies by geographical region. In this study, there is also a possibility that differences in malaria incidence between malnourished and healthy children were, partially, masked due to very high SMC and long-lasting insecticidal net coverage.

Several strengths of this study can be identified. Multiple, more established as well as more novel, nutritional measures were used and compared. Moreover, this study had a much larger sample size than most previous observational studies and malaria incidence was very high. There was also little loss to follow-up and few individuals had missing data. In this study malaria incidence was measured prospectively, allowing multiple events per person and the calculation of rates instead of risks.

Key limitations of this study are the potential presence of unmeasured confounding, variation in type of anthropometric measurements collected between years and variation in study size between years. Some unmeasured confounding might be present in this study, given that limited data on potential confounders was collected. Socio-economic status is likely to have a confounding effect on the relationship between malnutrition and malaria, because an association with both has been shown [36, 37], but this was not recorded. The variation in measurements recorded between years resulted in a larger sample size available for analyses regarding MUAC compared to stunting, wasting and underweight. Therefore, the findings related to MUAC are more robust than those for the other nutritional measures. However, also for stunting, wasting and underweight, the sample size was fairly large.

To reduce the risk of reverse causality, a prospective design was used determining the nutritional status of children at the start of the peak transmission season. It is however still possible that children have been exposed to malaria in previous transmission seasons or outside of the peak transmission season. This could mean that for some children their nutritional status is affected by previous malaria episodes.

Even though this study does not provide clear evidence about an association between malnutrition and malaria risk, it shows clearly that both conditions are causing a high disease burden. The use of SMC as a platform for nutritional interventions requires further investigation, and could have important impacts on nutritional status and malaria burden [17].

## Conclusion

Malnutrition was common in the two study areas in Burkina Faso and Mali and malaria incidence was high despite high SMC and long-lasting insecticidal net coverage. A high joint burden of malaria and malnutrition is likely to be found in a wide range of settings across the Sahel region. Despite the high burden of both malnutrition and malaria, the large study size, and the prospective study design, no strong evidence was found for an association between measures of nutritional status and clinical malaria incidence. However, an in-depth exploration of the potential effect of nutritional interventions on malaria control would still be valuable and could give important insights for reducing malaria burden in children.

## Supplementary Information

**Additional file 1: Figure S1.** Variation in prevalence of malnutrition (z-score below − 2) for MUAC and MUAC-for-age.

**Additional file 2: Table S1.** Association of baseline variables with low MUAC-for-age in two cohorts.

**Additional file 3: Table S1.** Association of baseline variables with low MUAC-for-age in two cohorts in Burkina Faso. **Table S2.** Association of baseline variables with stunting, wasting and underweight in 2016 in Burkina Faso.

**Additional file 4: Table S1.** Association of baseline variables with low MUAC-for-age in two cohorts in Mali. **Table S2.** Association of baseline variables with stunting, wasting and underweight in 2016 in Mali.

## Data Availability

The datasets generated and/or analysed during the current study will be archived on the LSHTM Data Compass repository and are available on reasonable request. Data will be archived on the LSHTM Data Compass institutional repository (http://datacompass.lshtm.ac.uk) for the purpose of ensuring long-term curation, preservation, and access. Given the nature of these data, we will ask users to sign a data sharing agreement. This is not intended to restrict access, but to ensure that requests are for ethical research purposes and that any analyses undertaken will not compromise the confidentiality of individual participants, and are not for commercial purposes.

## Abbreviations

MUAC: Mid-upper arm circumference
SMC: Seasonal malaria chemoprevention
DHS: Demographic and health survey
AZ: Azithromycin
SP: Sulfadoxine/pyrimethamine
AQ: Amodiaquine
RDT: Rapid diagnostic test
