# Supplementary figures and images for: Nutritional status in young children prior to the malaria transmission season in Burkina Faso and Mali, and its impact on the incidence of clinical malaria
Source: Malar J. 2021 Jun 22;20:274. doi: 10.1186/s12936-021-03802-2 (PMC8220741; doi:10.1186/s12936-021-03802-2)

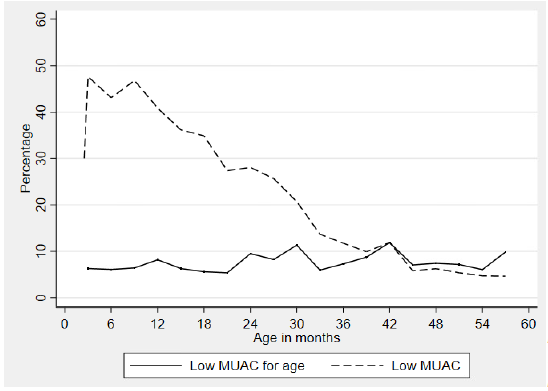

Supplement: Supplementary file 1 — Additional file 1: Figure S1. Variation in prevalence of malnutrition (z-score below − 2) for MUAC and MUAC-for-age. [file 12936_2021_3802_MOESM1_ESM.png]
