# Supplementary material for: Nutritional status in young children prior to the malaria transmission season in Burkina Faso and Mali, and its impact on the incidence of clinical malaria
Source: Malar J. 2021 Jun 22;20:274. doi: 10.1186/s12936-021-03802-2 (PMC8220741; doi:10.1186/s12936-021-03802-2)
Supplement: Supplementary file 2 — Additional file 2: Table S1. Association of baseline variables with low MUAC-for-age in two cohorts. [file 12936_2021_3802_MOESM2_ESM.docx]

# additional file 2

**MUAC-for-age**

*Table 1: Association of baseline variables with low MUAC-for-age in two cohorts.*

*Number and proportion with z<-2 are shown together with odds ratios and p-values. Likelihood ratio test p-values are presented to indicate a global measure of association.*

|  | **Low MUAC-for-age (2015)** | | | **Low MUAC-for-age (2016)** | | |
| --- | --- | --- | --- | --- | --- | --- |
|  | **Number (%)** | **Odds ratio** | **P-value** | **Number (%)** | **Odds ratio** | **P-value** |
| **Sex**  Boy  Girl | 1034 (10.6)  828 (9.0) | 1  0.79 (0.71-0.89) | 0.0001 | 162 (7.5)  144 (7.7) | 1  1.02 (0.79-1.32) | 0.86 |
| **Age in months**  3-12  13-24  25-36  37-48  48+ | 198 (7.0)  405 (9.3)  506 (12.1)  382 (9.6)  371 (10.4) | 1  1.49 (1.22-1.82)  2.07 (1.70-2.52)  1.55 (1.26-1.89)  1.72 (1.40-2.10) | <0.0001 | 36 (6.3)  55 (6.4)  83 (8.7)  73 (8.7)  59 (7.6) | 1  1.01 (0.64-1.61)  1.44 (0.94-2.23)  1.43 (0.92-2.23)  1.23 (0.78-1.95) | 0.22 |
| **Country**  Burkina Faso  Mali | 933 (9.8)  929 (9.9) | 1  1.01 (0.89-1.14) | 0.92 | 173 (8.4)  133 (6.8) | 1  0.77 (0.59-0.99) | 0.05 |
| **Intervention arm**  Placebo  AZ | 936 (9.9)  926 (9.8) | 1  0.98 (0.86-1.11) | 0.72 | 154 (7.7)  152 (7.6) | 1  0.97 (0.75-1.26) | 0.84 |
| **Distance to health facility**  <1 km  1-4 km  5-9 km  10+ km | 519 (8.9)  767 (10.6)  331 (9.7)  245 (10.0) | 1  1.22 (1.05-1.42)  1.11 (0.92-1.34)  1.13 (0.91-1.40) | 0.07 | 89 (6.9)  116 (7.9)  71 (9.5)  30 (6.1) | 1  1.17 (0.85-1.60)  1.45 (1.01-2.08)  0.87 (0.54-1.38) | 0.12 |
| **SP dose (mg/kg)**  <25  25-70  >70 | -- |  |  | 2 (7.1)  277 (7.2)  27 (21.3) | 1.04 (0.22-4.90)  1  4.06 (2.38-6.91) | <0.0001 |
| **AQ dose (mg/kg)**  <10  10-15  >15 | -- |  |  | 12 (1.6)  165 (7.0)  129 (14.2) | 0.20 (0.11-0.36)  1  2.36 (1.78-3.14) | <0.0001 |
