# Supplementary material for: Nutritional status in young children prior to the malaria transmission season in Burkina Faso and Mali, and its impact on the incidence of clinical malaria
Source: Malar J. 2021 Jun 22;20:274. doi: 10.1186/s12936-021-03802-2 (PMC8220741; doi:10.1186/s12936-021-03802-2)
Supplement: Supplementary file 3 — Additional file 3: Table S1. Association of baseline variables with low MUAC-for-age in two cohorts in Burkina Faso. Table S2. Association of baseline variables with stunting, wasting and underweight in 2016 in Burkina Faso. [file 12936_2021_3802_MOESM3_ESM.docx]

# Additional file 3

**Burkina Faso-specific results**

*Table 1: Association of baseline variables with low MUAC-for-age in two cohorts in Burkina Faso. Number and proportion with z<-2 is shown together with odds ratios and p-values. Likelihood ratio test p-values are presented to indicate a global measure of association.*

|  | **Low MUAC-for-age (2015)** | | | **Low MUAC-for-age (2016)** | | |
| --- | --- | --- | --- | --- | --- | --- |
|  | **Number (%)** | **Odds ratio** | **P-value** | **Number (%)** | **Odds ratio** | **P-value** |
| **Sex**  Boy  Girl | 509 (10.4)  424 (9.1) | 1  0.85 (0.74-0.99) | 0.03 | 82 (7.6)  91 (9.3) | 1  1.25 (0.89-1.75) | 0.20 |
| **Age in months**  3-12  13-24  25-36  37-48  48+ | 97 (6.6)  163 (7.6)  263 (12.4)  195 (10.0)  215 (11.6) | 1  1.20 (0.91-1.59)  2.19 (1.68-2.86)  1.68 (1.27-2.20)  2.01 (1.53-2.64) | <0.0001 | 18 (6.6)  31 (6.9)  46 (9.7)  43 (9.6)  35 (8.5) | 1  1.04 (0.54-1.98)  1.56 (0.84-2.88)  1.53 (0.83-2.85)  1.35 (0.71-2.55) | 0.39 |
| **Intervention arm**  Placebo  AZ | 477 (10.1)  456 (9.4) | 1  0.92 (0.79-1.07) | 0.26 | 87 (8.6)  86 (8.3) | 1  0.95 (0.67-1.34) | 0.77 |
| **Distance to health facility**  <1 km  1-4 km  5-9 km  10+ km | 147 (8.5)  586 (10.0)  181 (10.0)  19 (10.8) | 1  1.23 (1.00-1.52)  1.22 (0.94-1.57)  1.37 (0.77-2.43) | 0.26 | 38 (8.9)  91 (7.7)  42 (10.1)  <5 | 1  0.84 (0.55-1.29)  1.16 (0.70-1.92)  0.82 (0.17-4.05) | 0.49 |
| **SP dose (mg/kg)**  <25  25-70  >70 | -- |  |  | 1 (8.3)  154 (7.9)  18 (19.4) | 1.10 (0.12-9.82)  1  3.04 (1.61-5.75) | 0.003 |
| **AQ dose (mg/kg)**  <10  10-15  >15 | -- |  |  | 6 (1.6)  95 (8.0)  72 (14.6) | 0.18 (0.08-0.43)  1  2.03 (1.41-2.94) | <0.0001 |

Table 2: *Association of baseline variables with stunting, wasting and underweight in 2016 in Burkina Faso. Number and proportion with z<-2 is shown together with odds ratios and p-values. Likelihood ratio test p-values are presented to indicate a global measure of association.*

|  | **Stunted** | | | **Wasted** | | | **Underweight** | | |
| --- | --- | --- | --- | --- | --- | --- | --- | --- | --- |
|  | **Number (%)** | **Odds ratio** | **P-value** | **Number (%)** | **Odds ratio** | **P-value** | **Number (%)** | **Odds ratio** | **P-value** |
| **Sex**  Boy  Girl | 303 (28.1)  217 (22.0) | 1  0.69 (0.55-0.87) | 0.002 | 293 (26.8)  259 (25.8) | 1  0.96 (0.70-1.31) | 0.78 | 343 (31.8)  288 (29.2) | 1  0.85 (0.66-1.11) | 0.23 |
| **Age in months**  3-12  13-24  25-36  37-48  48+ | 46 (16.3)  137 (30.6)  134 (28.2)  112 (24.9)  91 (22.1) | 1  2.48 (1.62-3.80)  2.18 (1.43-3.32)  1.82 (1.19-2.79)  1.54 (1.00-2.37) | 0.0003 | 93 (32.9)  161 (35.9)  113 (23.8)  98 (21.8)  82 (20.0) | 1  1.23 (0.71-2.15)  0.44 (0.24-0.81)  0.36 (0.19-0.67)  0.30 (0.15-0.58) | <0.0001 | 84 (29.7)  165 (36.8)  153 (32.2)  121 (27.0)  108 (26.3) | 1  1.57 (0.99-2.47)  1.20 (0.77-1.89)  0.81 (0.51-1.29)  0.80 (0.50-1.27) | 0.009 |
| **Intervention arm**  Placebo  AZ | 273 (26.7)  247 (23.6) | 1  0.83 (0.66-1.04) | 0.10 | 283 (27.3)  269 (25.3) | 1  0.83 (0.60-1.16) | 0.27 | 332 (32.5)  299 (28.6) | 1  0.77 (0.58-1.01) | 0.06 |
| **Distance to health facility**  <1 km  1-4 km  5-9 km  10+ km | 108 (25.1)  281 (23.6)  119 (28.6)  12 (44.4) | 1  0.92 (0.69-1.22)  1.23 (0.87-1.73)  2.73 (1.09-6.81) | 0.03 | 92 (21.3)  326 (26.9)  132 (31.0)  <5 | 1  1.65 (1.06-2.57)  2.37 (1.36-4.11)  0.17 (0.02-1.41) | 0.005 | 108 (25.1)  362 (30.3)  155 (37.3)  6 (22.2) | 1  1.46 (1.01-2.09)  2.30 (1.46-3.64)  0.87 (0.24-3.18) | 0.004 |
| **SP dose (mg/kg)**  <25  25-70  >70 | 1 (7.7)  469 (23.9)  50 (53.8) | 0.21 (0.02-1.87)  1  4.59 (2.65-7.97) | <0.0001 | 0  466 (23.4)  86 (91.5) | --  1  206.7 (43.6-979) | <0.0001 | 0 (0.0)  538 (27.5)  93 (100.0) | *Not relevant* |  |
| **AQ dose (mg/kg)**  <10  10-15  >15 | 24 (6.5)  290 (24.2)  206 (41.5) | 0.20 (0.12-0.32)  1  2.44 (1.84-3.25) | <0.0001 | 12 (3.2)  264 (21.6)  276 (55.2) | 0.07 (0.03-0.16)  1  8.58 (4.94-14.88) | <0.0001 | 0  300 (25.0)  331 (66.7) | --  1  10.05 (5.68-17.8) | <0.0001 |
