# Supplementary material for: Nutritional status in young children prior to the malaria transmission season in Burkina Faso and Mali, and its impact on the incidence of clinical malaria
Source: Malar J. 2021 Jun 22;20:274. doi: 10.1186/s12936-021-03802-2 (PMC8220741; doi:10.1186/s12936-021-03802-2)
Supplement: Supplementary file 4 — Additional file 4: Table S1. Association of baseline variables with low MUAC-for-age in two cohorts in Mali. Table S2. Association of baseline variables with stunting, wasting and underweight in 2016 in Mali. [file 12936_2021_3802_MOESM4_ESM.docx]

# additional file 4

**Mali-specific results**

*Table 1: Association of baseline variables with low MUAC-for-age in two cohorts in Mali. Number and proportion with z<-2 is shown together with odds ratios and p-values. Likelihood ratio test p-values are presented to indicate a global measure of association.*

|  | **Low MUAC-for-age (2015)** | | | **Low MUAC-for-age (2016)** | | |
| --- | --- | --- | --- | --- | --- | --- |
|  | **Number (%)** | **Odds ratio** | **P-value** | **Number (%)** | **Odds ratio** | **P-value** |
| **Sex**  Boy  Girl | 525 (10.9)  404 (8.8) | 1  0.75 (0.63-0.88) | 0.0001 | 80 (7.4)  53 (6.0) | 1  0.78 (0.53-1.15) | 0.20 |
| **Age in months**  3-12  13-24  25-36  37-48  48+ | 101 (7.4)  242 (10.9)  243 (11.8)  187 (9.3)  156 (9.2) | 1  1.77 (1.34-2.36)  1.87 (1.41-2.48)  1.38 (1.03-1.84)  1.36 (1.00-1.84) | 0.0001 | 18 (6.0)  24 (5.8)  37 (7.7)  30 (7.7)  24 (6.5) | 1  0.97 (0.49-1.89)  1.32 (0.71-2.46)  1.29 (0.67-2.47)  1.08 (0.55-2.12) | 0.77 |
| **Intervention arm**  Placebo  AZ | 459 (9.7)  470 (10.1) | 1  1.08 (0.88-1.31) | 0.47 | 67 (6.7)  66 (6.9) | 1  1.00 (0.67-1.47) | 0.99 |
| **Distance to health facility**  <1 km  1-4 km  5-9 km  10+ km | 372 (9.1)  181 (13.1)  150 (9.3)  226 (10.0) | 1  1.44 (1.10-1.90)  1.03 (0.78-1.37)  1.07 (0.83-1.37) | 0.07 | 51 (5.9)  25 (8.6)  29 (8.7)  28 (6.0) | 1  1.56 (0.90-2.71)  1.55 (0.91-2.65)  1.03 (0.61-1.73) | 0.22 |
| **SP dose (mg/kg)**  <25  25-70  >70 | -- |  |  | 1 (6.3)  123 (6.5)  9 (26.5) | 1.02 (0.11-9.17)  1  6.94 (2.61-18.4) | 0.0005 |
| **AQ dose (mg/kg)**  <10  10-15  >15 | -- |  |  | 6 (1.5)  70 (6.1)  57 (13.9) | 0.22 (0.09-0.53)  1  2.79 (1.81-4.30) | <0.0001 |

Table 2: *Association of baseline variables with stunting, wasting and underweight in 2016 in Mali. Number and proportion with z<-2 is shown together with odds ratios and p-values. Likelihood ratio test p-values are presented to indicate a global measure of association.*

Table 4: *Association of baseline variables with stunting, wasting and underweight in 2016 in Mali. Number and proportion with z<-2 is shown together with odds ratios and p-values. Likelihood ratio test p-values are presented to indicate a global measure of association.*

|  | **Stunted** | | | **Wasted** | | | **Underweight** | | |
| --- | --- | --- | --- | --- | --- | --- | --- | --- | --- |
|  | **Number (%)** | **Odds ratio** | **P-value** | **Number (%)** | **Odds ratio** | **P-value** | **Number (%)** | **Odds ratio** | **P-value** |
| **Sex**  Boy  Girl | 326 (30.3)  213 (24.1) | 1  0.72 (0.58-0.89) | 0.003 | 195 (17.4)  122 (13.3) | 1  0.71 (0.54-0.93) | 0.01 | 263 (24.4)  167 (18.9) | 1  0.70 (0.56-0.89) | 0.004 |
| **Age in months**  3-12  13-24  25-36  37-48  48+ | 33 (10.9)  125 (30.2)  174 (36.1)  114 (29.2)  93 (25.1) | 1  3.90 (2.48-6.13)  5.18 (3.32-8.09)  3.69 (2.34-5.82)  2.91 (1.84-4.60) | <0.0001 | 69 (22.8)  87 (21.0)  61 (12.7)  42 (10.7)  50 (13.5) | 1  0.87 (0.57-1.31)  0.44 (0.29-0.69)  0.35 (0.22-0.57)  0.50 (0.31-0.79) | <0.0001 | 48 (15.8)  107 (25.9)  124 (25.7)  74 (18.9)  77 (20.8) | 1  1.96 (1.30-2.96)  1.96 (1.31-2.93)  1.28 (0.83-1.96)  1.46 (0.95-2.24) | 0.003 |
| **Intervention arm**  Placebo  AZ | 274 (27.5)  265 (27.5) | 1  1.00 (0.81-1.25) | 0.96 | 164 (15.9)  153 (15.2) | 1  0.93 (0.70-1.23) | 0.59 | 213 (21.4)  217 (22.5) | 1  1.08 (0.85-1.37) | 0.55 |
| **Distance to health facility**  <1 km  1-4 km  5-9 km  10+ km | 233 (26.8)  67 (23.1)  101 (30.3)  138 (29.6) | 1  0.81 (0.58-1.14)  1.20 (0.89-1.62)  1.16 (0.89-1.52) | 0.17 | 102 (11.2)  45 (14.8)  59 (17.2)  111 (23.1) | 1  1.42 (0.92-2.17)  1.70 (1.13-2.54)  2.58 (1.82-3.67) | <0.0001 | 145 (16.7)  64 (22.1)  89 (26.7)  132 (28.3) | 1  1.42 (1.00-2.03)  1.87 (1.34-2.60)  2.02 (1.51-2.72) | <0.0001 |
| **SP dose (mg/kg)**  <25  25-70  >70 | 1 (6.3)  517 (27.1)  21 (61.8) | 0.17 (0.02-1.36)  1  4.88 (2.27-10.5) | 0.0001 | 1 (5.6)  287 (14.4)  29 (85.3) | 0.34 (0.04-2.84)  1  53.6 (17.3-166) | <0.0001 | 0 (0.0)  396 (20.7)  34 (100.0) | *Not relevant* |  |
| **AQ dose (mg/kg)**  <10  10-15  >15 | 18 (4.5)  315 (27.3)  206 (50.1) | 0.11 (0.07-0.19)  1  3.04 (2.28-4.05) | <0.0001 | 21 (5.0)  159 (13.2)  137 (33.3) | 0.33 (0.20-0.54)  1  3.69 (2.68-5.08) | <0.0001 | 1 (0.3)  206 (17.9)  223 (54.3) | 0.01 (0.002-0.08)  1  6.13 (4.41-8.51) | <0.0001 |
